# Supplementary material for: Meru couples planar cell polarity with apical-basal polarity during asymmetric cell division
Source: eLife. 2017 Jun 30;6:e25014. doi: 10.7554/eLife.25014 (PMC5493435; doi:10.7554/eLife.25014)
Supplement: Supplementary file 1. — DOI: http://dx.doi.org/10.7554/eLife.25014.021 [file elife-25014-supp1.docx]

| Genotype A | Genotype B | P value | Summary |
| --- | --- | --- | --- |
| Figure 1I | | | |
| *meru^1^* | *meru^1^/Df* | > 0.9999 | n.s. |
| *meru^2^* | *meru^2^/Df* | 0.0381 | * |
| *ctrl* | *meru^1^* | < 0.0001 | *** |
| *ctrl* | *meru^2^* | 0.0052 | ** |
| *meru^1^* | *meru^2^* | < 0.0001 | *** |
| *meru^1^/Df* | *meru^2^/Df* | < 0.0001 | *** |
| *meru^1^* | *ubi-GFP/+; meru^1^* | 0.0031 | ** |
| *ubi-GFP/+; meru^1^* | *ubi-GFP-meru/+; meru^1^* | < 0.0001 | *** |
| Figure 1I' | | | |
| *meru^1^* | *meru^1^/Df* | 0.0186 | * |
| *meru^2^* | *meru^2^/Df* | 0.1885 | n.s. |
| *ctrl* | *meru^1^* | < 0.0001 | *** |
| *ctrl* | *meru^2^* | 0.1118 | n.s. |
| *meru^1^* | *meru^2^* | 0.0028 | ** |
| *meru^1^/Df* | *meru^2^/Df* | 0.0001 | ** |
| *meru^1^* | *ubi-GFP/+; meru^1^* | 0.0870 | n.s. |
| *ubi-GFP/+; meru^1^* | *ubi-GFP-meru/+; meru^1^* | 0.0310 | * |
| Figure 3I | | | |
| *nubG4/baz^IR^* | *nubG4/baz^IR^; meru^1^* | < 0.0001 | *** |
| *nubG4/UAS-GFP; meru^1^* | *nubG4/baz^IR^; meru^1^* | < 0.0001 | *** |
| *nubG4/pins^IR^* | *nubG4/pins^IR^; meru^1^* | < 0.0001 | *** |
| *nubG4/UAS-GFP; meru^1^* | *nubG4/pins^IR^; meru^1^* | < 0.0001 | *** |
| *nubG4/Gα_i_^IR^* | *nubG4/Gα_i_^IR^; meru^1^* | < 0.0001 | *** |
| *nubG4/UAS-GFP; meru^1^* | *nubG4/Gα_i_^IR^; meru^1^* | < 0.0001 | *** |
| Figure 3-figure supplement 1D | | | |
| *meru^1^* | *dsh^1^;; meru^1^* | < 0.0001 | *** |
| *dsh^1^* | *dsh^1^;; meru^1^* | < 0.0001 | *** |
| *meru^1^* | *dsh^1^;; meru^1^/+* | < 0.0001 | *** |
| *dsh^1^* | *dsh^1^;; meru^1^/+* | 0.6210 | n.s. |
| Figure 3-figure supplement 1D' | | | |
| *meru^1^* | *dsh^1^;; meru^1^* | < 0.0001 | *** |
| *dsh^1^* | *dsh^1^;; meru^1^* | < 0.0001 | *** |
| *meru^1^* | *dsh^1^;; meru^1^/+* | 0.0280 | * |
| *dsh^1^* | *dsh^1^;; meru^1^/+* | 0.5955 | n.s. |
| Figure 3-figure supplement 1E | | | |
| *nubG4/UAS-GFP; meru^1^* | *nubG4/fz^IR^; meru^1^* | < 0.0001 | *** |
| *nubG4/UAS-GFP; meru^1^* | *nubG4, UAS-fz/+; meru^1^* | < 0.0001 | *** |
| *nubG4/UAS-GFP; meru^1^* | *nubG4, UAS-arm/+; meru^1^* | 0.0641 | n.s. |
| Figure 6-figure supplement 1F | | | |
| *UAS-GFP/+; neurG4, meru^1^/meru^1^* | *UAS-baz-GFP/+; neurG4, meru^1^/meru^1^* | < 0.0001 | *** |
| *UAS-baz-GFP/+; neurG4, meru^1^/meru^1^* | *UAS-baz-GFP/+; neurG4/+* | 0.0350 | * |
| Figure 6-figure supplement 1F' | | | |
| *UAS-GFP/+; neurG4, meru^1^/meru^1^* | *UAS-baz-GFP/+; neurG4, meru^1^/meru^1^* | < 0.0001 | *** |
| *UAS-baz-GFP/+; neurG4, meru^1^/meru^1^* | *UAS-baz-GFP/+; neurG4/+* | > 0.9999 | n.s. |
